# Immunosenescence: Driver of Cardiac Aging

**DOI:** 10.14336/AD.2025.0590

**Published:** 2026-08-08

**Authors:** Simeng Zhu, Guofang Xia, Yifan Shi, Congfeng Xu

**Affiliations:** Department of Cardiology, Shanghai Jiaotong University School of Medicine Affiliated Sixth Hospital, Shanghai, China

**Keywords:** immune cells, cellular senescence, aging, inflammaging/chronic inflammation

## Abstract

Immunity is essential for host’s survival, and aging of immune system (immunosenescence) may compromise host immunity, leading to chronic, low-grade inflammation, consequently contributing to aging and age-related diseases, like cardiovascular diseases, neurodegenerative diseases. Although with a strong reserve capacity, hearts of the elderly manifest structural and functional changes, including left ventricular hypertrophy, left ventricular enlargement, interstitial fibrosis and diastolic dysfunction. The senescent immune cells undergo decline in antigen processing and presentation capacity, reduced phagocytosis, increased inflammatory phenotype (SASP), and limited specificity. Through autocrine and paracrine style, these senescent cells lead to cardiac aging and cardiac diseases, including atherosclerosis, cardiomyopathy and heart failure. Senotherapeutics development on eliminating or modulating senescent immune cells to alleviate cardiac aging and cardiovascular diseases is also covered in this review.

## Introduction

Currently, our society is undergoing a rapid aging process, with the population over 60 years doubled (2.1 billion) and that of over 80 years tripled by 2050 (426 million), according to World Health Organization (WHO; www.who.int/news-room/fact-sheets/detail/ageing-and-health). Driven by environmental and genetic factors, aging exhibit an array of hallmarks, such as cellular senescence, chronic inflammation, mitochondrial dysfunction, genomic instability, consequently manifested by age-related diseases, including cancers, neurodegenerative diseases, metabolic disorders, and cardiovascular diseases [1]. These age-related diseases contribute to morbidity and mortality of senior population [2].

Multiple studies have demonstrated that the elderly displayed an active state of systemic inflammation, featured by increased circulating levels of proinflammatory cytokines, such as TNF-α and IL-6, and localized tissue inflammation [3]. This kind of low-grade inflammation correlates with aging (inflammaging) and usually leads to tissue and organ injury [4]. Meanwhile, the older individuals also more likely show a presence of autoantibodies [5]. Naturally, age-related diseases, such as cardiovascular diseases and degenerative diseases, have clearly shown evidence of systemic or tissue inflammation [6].

During aging, virtually all cells and organs undergo progressive functional impairment and homeostasis disorder. Undoubtedly, immune system has no exception, with numerous and profound changes with aging (immunosenescence) [7]. As a surveillance system, the immune system ceaselessly monitors the presence of exogenous and endogenous threats, such as pathogen-associated molecular patterns (PAMP), damage-associated molecular patterns (DAMP), and aims to eliminate the stresses and maintain homeostasis [8]. While immunosenescence diminishes the responses against pathogen invasion, autoimmunity, as well as chronic disorders, such as degenerative and cardiovascular diseases [9].

As the leading cause of mortality in senior population, cardiovascular diseases are commonly regarded as age-related disorders. A growing body of evidence has linked immunosenescence with cardiac aging and cardiovascular dysfunction [10]. Indeed, cardiac aging usually comes along with persistent low-grade inflammation, commonly termed as 'cardiac inflammaging'. The causal role of inflammation for cardiovascular disease was substantiated by the CANTOS trial (Canakinumab Anti-Inflammatory Thrombosis Outcomes Study), in which the risk of cardiovascular events was largely reduced by IL-1β inhibition, particularly in participants with elevated IL-6 levels [11-13].

In the current review, we first describe the cellular and molecular changes during immunosenescence, then discuss the implications of immunoscence in diverse cardiovascular pathological processes and lastly explicate how regulation of immunosenescence processes could be captalized on therapeutic intervention of cardiovascular pathologies.

### Immunosenescence

Being responsible for host defense to pathogens, supervision on cellular aberration, and homeostasis maintenance, immune system plays paramount role during organism development, growth, and health. As most immune cells are derived from hematopoietic stem cells (HSC) in bone marrow, the differentiation capability of HSC is critical for host immunity [10]. During the aging process, self-renew potential of HSC is considerably reduced, and the differentiation balance between myeloid and lymphoid lineage is also impaired, with a bias towards the myeloid lineage. This bias could be due to massive clonal expansion and persistence of antigen-selected cells for decades, which impose enormous proliferative pressure on immune cells, making the immune system highly susceptible to the aging process [14-16]. With this progressive deterioration of protective immunity, the older individuals become more vulnerable to infection, malignancies, autoimmune diseases, and many other age-related diseases, such as cardiovascular diseases [17]. This phenomenon in which the immune system gradually declines with age is immunosenescence [18]. With impaired immune surveillance, more inflammatory response and less efficient resolution of inflammation, immunosenescence is coupled with amplifying chronic inflammatory state associated with inflammaging [15]. Inflammaging, featured by chronic systemic inflammation, impairs immune function additionally, and plays a vital role in host aging process.

### Adaptive immune system senescence

Lymphocytes, derived from common lymphoid progenitor cells, are more susceptible to change with aging, compared with myeloid cells. Upon aging, production of naïve T cells is reduced, and the cytotoxic CD8^+^ T cells exhibit a drastic decrease in the elderly, while CD4^+^ T cells are less affected [19]. However, the CD4^+^ T cells acquire cytotoxic phenotype and more pro-inflammatory in senior population [20], releasing various pro-inflammatory cytokines, such as interleukins and tumor necrosis factor (TNF) [21]. In addition, the Treg subpopulation in CD4^+^ T cells are not only considerably less, but also with compromised suppressive capabilities in elderly [22]. While activated CD8^+^ T cells from aged individuals display features of cellular senescence with differentiated memory phenotype [23]. Aging T cells possess less antigen specificity, especially for CD8^+^ cells, which worked in an antigen-independent style, instead of a typical TCR-mediated activity [24]. Currently, there are no commonly accepted biomarkers to characterize senescent T cells, although CD81^+^CD28^-^ T cells were regarded as senescent lymphocytes. Increased expression of CD57 or CD153 and reduced CD27 have been also proposed as biomarkers of senescent-like T cells [25, 26].

Also derived from common lymphoid progenitor cells, B lymphocytes, especially those expressing CD28, decrease significantly with aging [27-29]. Two age-related B cell subsets have been identified [30, 31]: Aged-associated B cells (ABC) in spleen and bone marrow, and adipose B cells (AAB) in fat associated lymphoid clusters. Another lymphoid lineage cells, natural killer (NK) cells, also increase in the peripheral blood with healthy aging [32].

### Innate immune system senescence

Innate immune cells, such as neutrophils, macrophages, and dendritic cells (DC), are derived from myeloid lineage. Most innate immune cells are increased in the elderly, with prominent dysfunction, such as decline in antigen processing and presentation capacity, reduced macrophage chemotaxis and phagocytosis, increased peroxide and cytokine/chemokines production, especially pro-inflammatory cytokines [33-36], contributing to systemic and microenvironmental aging, e.g. "inflammaging". The aging-related proinflammatory state is closely associated with increased levels of proinflammatory mediators, such as IL-6, TNF-α, IL-1α, CRP, interferons (IFN-β1 and α), which were termed senescence-associated secretory phenotype (SASP). Ranging from cytokines, chemokines, growth factors and extracellular matrix (ECM) proteases, SASP is highly heterogeneous [37-40], and are widely used as readouts to assess aging/inflammaging. Notably, inflammatory SASP-producing senescent cells can cause substantial pathological damage and lead to aging-related phenotypes [41].

Senescent macrophages are the main cells with high levels of SA-β-Gal staining and SASP production [42], and play a critical role in maintaining chronic low-grade inflammation in age-related diseases [43, 44]. These highly senescent macrophages could promote itself senescence (autocrine) and the senescence of the surrounding cells (paracrine) [45-47], thus propagating extensive inflammation [48]. Cardiac macrophages from aged mice display impaired phagocytosis and the resolution of inflammation [49, 50], probably resulting from reduced expression of TIM-4 receptor, which is responsible for phagocytosis [51]. The impaired phagocytosis also aggravates senescence due to the accumulation of senescent cells [52]. Combined with reduced production of specialized pro-resolving mediators (SPM) [53], senescent macrophages could release more SASP and induce more neutrophile infiltration [54], leading to increased generation of the SASP and exacerbated inflammation.

**Figure 1.**
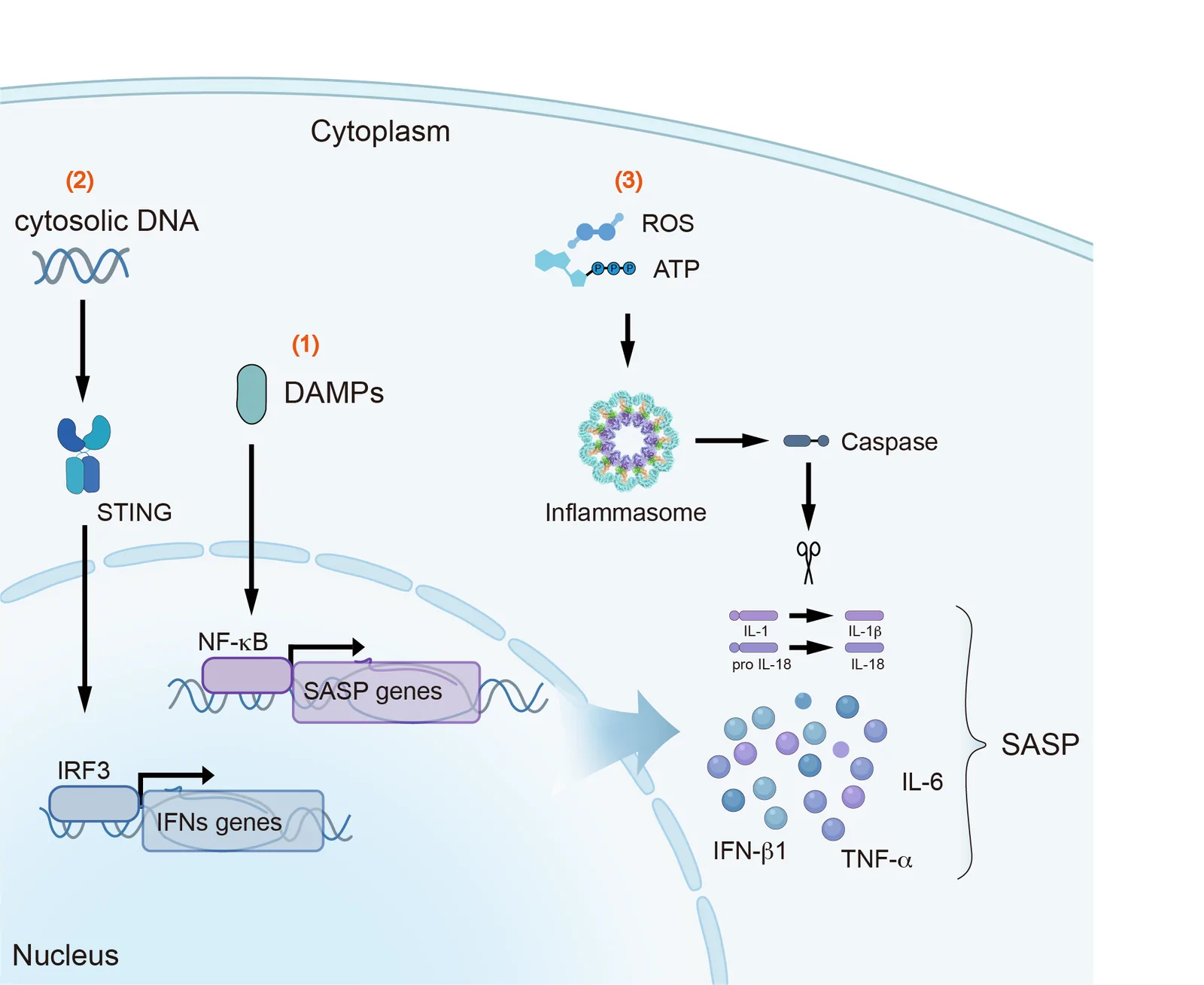
**Regulation of SASP**. As a dynamic process during aging, initiation and production of senescence-associated secretory phenotype (SASP) is fine-tuned. With aging, (1) intracellular and extracellular damage-associated molecular patterns (DAMP), such as high mobility group box 1 (HMGB1), S100 proteins, and heat shock proteins (HSP), induce SASP genes via nuclear factor-κB (NF-κB) activation. (2) Meanwhile, DNA sensor cyclic GMP-AMP synthase (cGAS) and the adaptor stimulator of interferon genes (STING) were also activated by cytosolic DNA leaked from mitochondria and nuclear, inducing SASP genes via interferon regulatory factor (IRF) 3 pathways. In addition, (3) inflammasome was also activated by ROS and ATP, leading to production of IL-1β and IL-18. These activated SASP genes contribute to production and releasing of many core SASP, such as proinflammatory tumor necrosis factor (TNF)-α, interleukin-6 (IL-6), interferon b (IFN-β). The SASP works in a paracrine and autocrine style during aging process.

### Core Inflammatory Pathways in Immunosenescence

Suppressed autophagy/mitophagy, increased ROS production, and downregulated DNases, resulted in cytosolic accumulation of nuclear and mitochondria DNA in senescent immune cells, activating chronic sterile inflammation. The leaked nuclear and mitochondria DNA stimulates DNA sensor cGAS and the adaptor protein STING, which activate NF-κB and IRF-3 pathway, leading to SASP initiation and execution (Fig. 1) [55, 56]. The activation of cGAS-STING pathway is critical for SASP production, as cGAS-STING deficiency leads to less proinflammatory SASP production and mitigates immunosurveillance on senescence *in vivo*. Loss-of-function mutations in the STING gene (R293Q) could antagonize aging-related diseases, especially in subjects of chronic lung diseases [57], while gain-of-function mutations are associated with severe inflammation [58]. The mammalian target of rapamycin (mTOR) pathway is also vital for promoting SASP production, through stimulating NF-κB pathway, IL-1α production, and the serine/threonine kinase MK2 [59]. Meanwhile, released DAMP (nuclear and mitochondria DNA, et al) and ROS in senescent cells due to aberrant autophagy and mitophagy activate NLRP3 inflammasome, leading to production of IL-1β and IL-18, which play critical role in aging and age-related diseases, such as atherosclerosis and metabolic syndrome. For example, NLRP3 inflammasome activation plays critical roles in cardiovascular aging through controlling the release some critical SASP components [60-62]. And *Nlrp3* deficiency or inhibition alleviates cardiac inflammaging, myocardial remodeling and HF development, improves cardiac function, and atrial fibrillation shortening in aged mice by suppressing inflammation and cytokine production, such as IL-1β and IL-18 [60, 63-66]. IL-1β could lead to TGFB1/TGF-β1 pathway in LV, and induces excessive collagen I deposition, thus favoring cardiac remodeling, LV hypertrophy, interstitial fibrosis, while CASP1-mediated cell death in cardiomyocytes [65, 67-69]. Furtherly, a recent study found that somatic NAP1L1 mutation (D349E) destabilizes nucleosome formation, causing DNA leaking into the cytoplasm and activating cGAS-STING pathway, which promotes the release of inflammatory mediators and leads to cardiac hypertrophy [70]. In addition, STING pathway activation also promotes the phosphorylation of STAT1 and the expression of NALP3, causing more production of pro-inflammatory cytokines, involving in vascular calcification of multiple cardiac disorders [71].

Multiple transcriptomics and proteomics datasets based on diverse senescence models also support the strong correlation between the senescence and the overexpression of various complement components [72, 73], and our analysis based on RNA-seq also showed boosted intracellular complement activation in senescent macrophages [49]. While the pathophysiological mechanisms remain to be revealed and is interesting to explore further.

Recent studies supported that immunosenescence act as a primary driver for systemic senescence and age-related diseases. Deficiency of ERCC1, a crucial DNA repair protein, in hematopoietic cells, leads to DNA damage and cellular senescence in multiple immune cell populations, including follicular T helper cells and NK cells. The mice with ERCC1 deletion demonstrate generally accelerated aging, such as the increased expression of p16 and p21 in non-lymphoid organs [74]. Similarly, deletion of TFAM, a key regulator of induction of mtDNA replication and transcription, in T lymphocytes leads to premature CD4^+^ T cell aging, resulting accumulation of circulating proinflammatory cytokines, which resembles the chronic inflammation typically associated with aging. This type of immunosenescence instigates various aging-related features, including cardiovascular, cognitive, metabolic, physical, and cardiovascular alterations, which together result in premature death [35].

To make the situation more complicated, immunosenescence may have beneficial effect on some age-related disorders. For example, higher levels of IFN-γ from NK or monocytes in blood plasma have protective effect against cognitive deterioration in elderly populations [75]. Natural IgM derived from aged B1 cells shows somewhat protective effects for infection, such as *Streptococcus pneumoniae* infection [76].

## Cardiac Aging

### Mechanisms of cardiac aging

With a strong reserve capacity, human hearts are rather resistant to aging and typically show no severe dysfunction among the healthy senior population. However, even in healthy elderly, age-related alterations could be easily detected, such as left ventricular hypertrophy, left ventricular enlargement, even interstitial fibrosis with some diastolic dysfunction. Compared with 4-month old mice, the hearts from aged mice (18-month old) showed elevated SA-β-galactosidase activity, a major marker of cell senescence, as well as cardiomyocyte hypertrophy and fibrosis [77]. Indeed, there is evident correlation between aging and increased risk of cardiovascular diseases, including heart failure with preserved ejection fraction (HFpEF), and electrical conduction system disorders, with distinct differences between male and female subjects [78].

### Cardiac macrophages in cardiac aging

Heart is a high energy-demanding organ, predominantly relying on mitochondrial oxidative phosphorylation for ATP production [79], and that is maybe why mitochondria compromise nearly one-third of the volume of adult cardiomyocytes [80]. Mitochondrial function declines with aging [9, 81], and this dysfunction has been shown to be a major cause of cardiac disease [82], especially for cardiomyocytes. Aging-related accumulation of damaged mitochondria and excessive mitochondrial ROS production result in DNA damage and telomere dysregulation, which trigger cellular senescence in cardiomyocytes independent of telomere length [81, 83, 84]. During the process, immune cells have been shown to play critical roles (Fig. 2), although the immunological senescent profiling for myocardium and vasculature is distinct (Table 1). Macrophages, especially cardiac resident macrophages, are the predominant immune cell population in the heart physiologically [85-90]. These cardiac macrophages are embryonically derived and are indispensable to heart development. Additionally, the cardiac macrophages also maintanin homeostasis, prevent fibrosis and stimulate angiogenesis in response to pressure overload [91, 92], probably by eliminating damaged mitochondria to ensure mitochondrial and cardiomyocyte fitness. Either cardiac macrophage depletion or Mertk, a phagocytic receptor, deficiency led to defective elimination of mitochondria from the myocardial tissue, impaired autophagy, accumulation of anomalous mitochondria in cardiomyocytes, metabolic alterations, activation of the inflammasome and ventricular dysfunction during aging [93].

**Figure 2.**
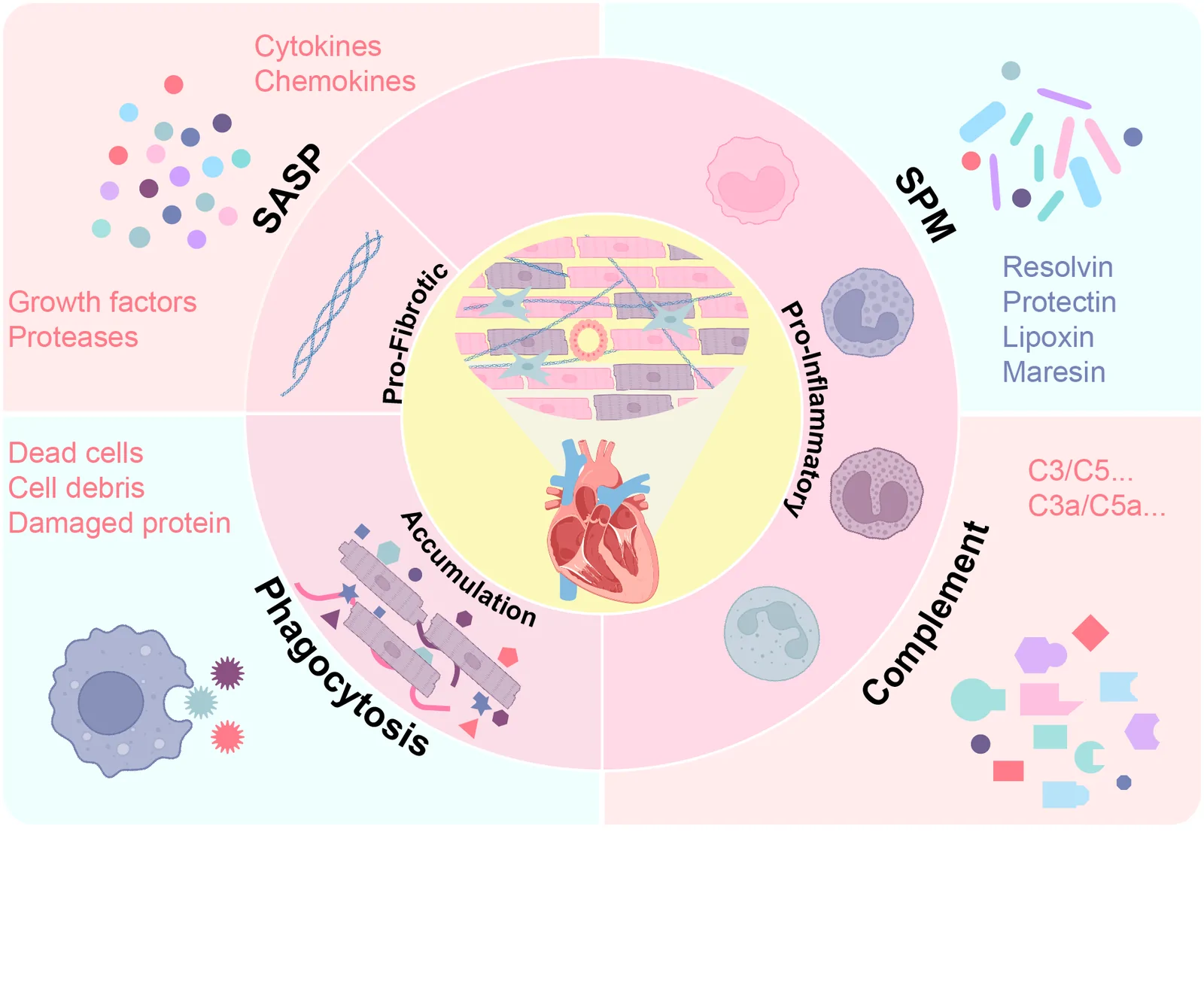
**Effect of immunosenescence on cardiac aging**. Immunosenescence leads to dysfunction of immune cells, such as increased inflammatory phenotype and SASP production, inappropriate activation of complement cascade, and reduced phagocytosis. Pro-inflammatory phenotype (mainly cytokines and chemokines) in most immune cells is manifested with chronic low-grade inflammation (inflammaging), resulted from excessively activated inflammation and impaired production of specialized pro-resolving mediators (SPM), is prominently present in aging hosts, and actively involved in aging process. Some SASP, especially matrix metalloproteinases (MMP), is pro-fibrotic, and contribute to fibrosis, leading to structural and function decline of cardiac aging. While limited phagocytosis, especially in macrophages, leads to accumulation of dead cells and cell debris, releasing multiple DAMP, which are usually both pro-inflammatory and pro-fibrotic.

Upon aging, resident macrophages are progressively replaced by recruited monocyte-derived macrophages [94]. Distinct from embryonically derived cardiac macrophages, the recruited monocytes/macrophages express CCR2, and might cause cardiovascular damage under malignant stimuli, such as ischemic injury, hypertension, and hyperlipidemia [92]. With aging, the reduced phagocytosis of these macrophages contributes to increased generation of the SASP, promoting inflammation and the development of age-related diseases [44, 95]. The SASP from senescent cardiac macrophages include various pro-inflammatory and pro-fibrotic cytokines, which impair the heart’s contractile and diastolic functions, and conduce to the remodeling of left ventricle [96], ultimately leading to various forms of heart failure. Additionally, these SASP also lead to endothelial damage, vascular remodeling, and contribute to atherosclerosis [97, 98]. We also observed evident increase in pro-inflammatory and pro-fibrotic mediators, such as TGF-β in senescent cardiac macrophages, with a significant upregulation of TGF-β-TGFβR1 crosstalk between CRMs and fibroblasts, which potentially promotes age-related cardiac fibrosis and remodeling in aged mice [49]. Additionally, senescent macrophages can contribute to other processes that are known to contribute to HFpEF, such as oxidative stress, fibrosis, and vascular stiffening [99]. In aged C57BL/6 mice (both sexes), SASP components, including TNF-α, MIP-1α, eotaxin, TNF-α, IL-1β, and IL-6, were found to be increased, and associated with altered function and structure of the heart and frailty [100], while anti-inflammatory implement has protective effect for cardiac aging.

**Table 1:** Senescent profiling for myocardium and vasculature.

	Myocardium	vasculature	Ref
**Gender bias**	Female	Male	
**Inflicting cells**	Cardiomyocyte, fibroblast	Endothelial cell, VSMC	[78, 171]
**Immune cells**	Resident cardiac macrophages	Adipose tissue, Recruiting cells: Perivascular infiltrating monocytes and macrophages, CD4^+^ T, CD8^+^ T, ABC	[172-176]
**SASP**	IL-6, IL-1β, TNF-α, CRP, IFN-γ, TGF-β, ROS	IL-1, TNF-α, IFN-γ, Adipokines, TGF-β, RNS, ROS, RSS	[177-179]
**Signaling pathways**	NF-κB, cGAS-STING	NF-κB, Inflammasome	
**Manifestation**	Cardiomyocyte hypertrophy, Cardiac fibrosis	Vascular diameter·, Thickening of arterial wall	[5, 49, 126]

Abbreviation: VSMC, Vascular smooth muscle cell; ABC, Age-associated B cell; IL, interleukin; TNF, tumor necrosis factor; CRP, C-reaction protein; IFN, interferon; TGF, transforming growth factor; ROS, reactive oxygen species; RNS, reactive nitrogen species; RSS, reactive sulfur species; NF-kB, nuclear factor kappa-light-chain-enhancer of activated B cell; cGAS-STING, cyclic GMP-AMP synthase - stimulator of interferon genes.

Cardiac resident macrophages are critical to maintain cardiac impulse conduction by connexin 43, which facilitate myocardial intercellular communication through gap junctions, while connexin 43 deletion in macrophages or congenital lack of macrophages leads to prolonged atrioventricular conduction and contributes to cardiomyocyte senescence [101, 102]. Channels such as Kv1.3, Kv1.5, and Kir2.1 may contribute to ion currents [103]. And amphiregulin produced by cardiac macrophages is essential to control connexin 43 phosphorylation and translocation in cardiomyocytes [104]. While the conductive microenvironment can promote the expression of Connexin 43 in CCR2^-^ CRMs to regulate paracrine signaling for MI repair [105].

Our analysis based on RNA sequencing of cardiac macrophages from aged mice (20 months old) revealed multiple genes associated with cardiomyopathy and fibrosis increased considerably [49, 106], highlighting the crucial role of macrophages for cardiomyocyte senescence and cardiac diseases. Besides macrophages, mast cells also play essential roles for cardiomyocyte hypertrophy and senescence [107], and depletion of mast cells reduces cardiomyocyte contractility and impaires post-ischemia cardiac function [108].

### Lymphocytes in cardiac aging

With aging, the heart-draining lymph node and myocardial T cells underwent clonal expansion and up-regulated pro-inflammatory transcription signature, marked by an increased interferon-g (IFN-γ) production [109]. Among them, more CD4^+^CD44^high^CD62^low^ T cells (activated or effector-memory) were observed, with pronounced Treg:Tcov ratio shift in aged mice, suggesting a dynamic change of T cell populations in age-related cardiac inflammation and dysfunction [110], and adoptive transfer of T cells from old to young mice could induce mild cardiac dysfunction [110], as well as cardiac inflammation typically observed at an old age [111]. Even transfer of adult T cells to neonates also induces local inflammation and impair cardiac regenerative capacity [112]. Deletion of TFAM, a mitochondrial transcription factor responsible for mtDNA stabilization and replication, induces T cell senescence, with prominent chronic inflammation and cardiac aging [35]. How these T cells were primed during the cardiac aging process remain elusive, with a reasonably speculation that cardiomyocyte components could be served as auto-antigens and results in an autoreactive immune response [22, 110], consistent to the study that MHCII^+^ resident macrophages could present CM components to T cells and results in priming them in the absence of injury [113].

### Cardiovascular Diseases

Atherosclersosis is the major pathological processes of age-associated cardiovascular diseases and can lead to most ischemia disorders, and heart failure. Abundant studies have illustrated that atherosclerosis is primarily associated with immunosenescence and inflammation [114]. Immunosenescence and the pro-inflammatory SASP are found in the elderly and in atherosclerotic subjects [98]. Immune cells ditectly interact with endothelial cells, which expose them to senescence-induced stimuli, and play prominent roles in atherosclerosis, while fibroblasts are essential for fibrosis, which is a hallmark in cardiovascular aging and various cardiovascular diseases. Several SASP factors, such TGF-β2 and Growth differentiation factor (GDF)15 were identified in human and murine cardiomyocytes senescence [115], probably through activating fibroblasts and its senescence. There are another two TGF-β family members, Activin and Growth differentiation factor (GDF)-11, have been found to play essential roles during cardiac aging and cardiovascular diseases [116, 117]. For example, activin is positively correlated with cardiac aging and heart failure [116], and forced expression of activin A promotes degradation of SERCA2a and lead to contractile dysfunction and heart failure [116], while deletion of type II receptors for activin improved cardiac function in aged mice [116]. Replenishing circulating GDF-11 attenuates cardiac hypertrophy in aged mice [117], highlighting the critical roles of SASP in age-related cardiac dysfunction.

### Atherosclerosis

As the fundamental pathology of cardiovascular diseases, atherosclerosis has been commonly regarded as an inflammatory disorder. Atherosclerosis could be seen in youth and earlier, and manifests progressively upon aging [69]. During the process, macrophages infiltrate into subintima and phagocytose modified lipid, leading to development of foam cells, which contribute to plaque formation and vascular remodeling. During the development, hallmarks of chronic inflammation, such as IL-6, TNF-α, and multiple chemokines are strongly associated with the pathology. Circulating biomarkers of inflammation serve as prognostic indicators for the progression of atherosclerosis with its cardiovascular complications, and inflammation evidently further drives secondary events of disease progression [118]. CANTOS has shown that targeting inflammation has significant therapeutic effect on atherosclerotic diseases [11].

Growing evidence showed that cellular senescence is critical for atherosclerosis. Multiple senescence biomarkers, such as P16/INK4A, P53, P21, and increased activity of SA-β-Gal, have been found in multiple stages of atherosclerotic plaques, and relate to the severity of atherosclerosis [119-122]. In LDL receptor deficient (*Ldlr^-/-^*) mice, high amount of SA-b-Gal- and p16-positive endothelial cells, vascular smooth muscle cells and macrophages have been accumulated in atherosclerotic plaques, while depletion of p16-positive cells in both p16-3MR and INK-ATTAC transgenic mice could reduce plaque formation and progression [119].

Transmission electron microscopy analysis of the early atherosclerotic lesion revealed a high accumulation of senescent macrophages in the fatty-streak lesions, while the elastic fibers remained intact [123], and these senescent foamy macrophages express high level of chemokines and adhesion molecules, such as VCAM1 and MCP1, resulting more release of SASP, such as IL-1α and TNF-α, and matrix metalloproteinases (MMP)-3 and MMP-13 [42]. These SASP components from macrophages work on endothelial cells and smooth muscle cells to accelerate the atherosclerotic process probably through a paracrine mechanism [42, 124]. The induced endothelial dysfunction, a hallmarker of vascular aging, hinders vasodilation and the repair capacity of the vasculature [125], increasing vascular stiffness and remodeling, luminal dilation[126-128].

Meanwhile, impairment of the vascular endothelium results in more macrophage diapedesis and deposition of low-density lipoprotein (LDL) particles, favoring LDL oxidization and an inflammation storm [129]. To make things worse, senescent macrophages express I ncreased arachidonate 15-lipoxygenase (ALOX15), which promotes production of SPM such as 9/13-HODE and suppresses inflammation resolution [130]. Besides pro-inflammatory cytokines, the senescent macrophages release more MMP, such as MMP9 and MMP7, degrading the matrix, and making the atherosclerotic plaques unstable, thus promoting atherosclerosis development [42, 131, 132]. Atherosclerotic mice model based on aged *Ldlr^-/-^* mice showed upregulated *Mmp*2 and *Mmp*9 expression and activity in atherosclerotic lesions, which promote macrophage infiltration [133], highlighting the critical role of SASP from macrophages for plaque instability and atherosclerosis development.

### Cardiomyopathy and heart failure

As a hallmark for cardiac aging, cardiomyocyte hypertrophy and consequent cardiomyopathy, especially dilated cardiomyopathy, are very common among senior people [134]. Heart failure with preserved ejection fraction (HFpEF) is another clinical syndrome associated with aging process, and gradually rises with age, up to around 20% in adults over 75 [135]. HFpEF is more strongly associated with aging, and systemic inflammatory comorbidities relative to HF with reduced ejection fraction (HFrEF). Wealthy evidence has demonstrated the critical role of immune dysfunction and inflammaging for these disorders.

Diastolic dysfunction due to LV and arterial stiffening serve as a predominant characteristic of HFpEF, and both diastolic dysfunction and ventricular-arterial stiffening have been identified as potential results from chronic low-grade pro-inflammatory state [136], while there exist much lower circulating inflammatory cytokines, such as IL1RL1 and CRP, in HFrEF patients [137]. A featured systemic chronic inflammation characterized by elevated oxidative stress has been found to be commonly present in elderly HFpEF and cardiomyopathy patients [138]. The oxidative stress-induced inflammation contributes to interstitial fibrosis and cardiac myocyte stiffening, which appears to be an underlying mechanism for heart failure. Diverse inflammatory signaling pathways, such as NF-κB, inflammasome, and cGAS-STING, are involved in the pathogenesis of cardiomyopathy and HFpEF. Transgenic mice with cardiomyocyte-specific constitutively active IKK2 (IKKβ, a catalytic component of NF-κB) were more susceptible to inflammatory dilated cardiomyopathy and heart failure, while dominant-negative IkB transgenic mice were resistant to the development of disease [139, 140]. Similarly, cardiac-specific overexpression of TNF-α induces dilated cardiomyopathy in even young mice [141], while NLRP3 inflammasome deficiency protected mice from age-related cardiac structural and functional alterations, including dilated cardiomyopathy [63]. In addition, inhibition of NLRP3 inflammasome activation attenuated the development of cardiac dysfunction [11, 142]. NAP1L1, a nucleosome assembly protein, has been found to be a predictive biomarker for age-related disorders, such as Alzheimer's disease [143]. These studies highlight the excessive inflammatory response for the development of cardiomyopathy and heart failure.

IL-17A, predominately secreted by activated Th17 cells, drives cardiomyocyte apoptosis following cardiac ischemia/reperfusion [144], probably through stimulating CCL2 expression in cardiomyocyte [145], while inhibiting T-cell activation could prevent pressure overload induced cardiomyocyte apoptosis and hypertrophic growth and heart failure [146]. Th17/Treg balance in heart was also critical for cardiac homeostasis, and adoptive transfer of CD4^+^CD25^+^ regulatory T (Treg) cells led improved Ang II-induced cardiac injury and fibrosis [147].

## Atherosclerosis

Senolytics and Senotherapeutics

Cellular senescence can be beneficial or detrimental for organism health [148]. However, senotherapeutics has been widely developed for aging and age-related disorders, including cardiac aging and age-related cardiovascular diseases. Senotherapeutics mainly include senolytic and senomorphic methods. Senolytics aim to eliminate senescent cells, whereas senomorphics modulate the properties of senescent cells without eliminating them [149]. Both senolytics (elimination of senescent cells) and senomorphics (modulation of properties of senescent cells) are largely regarded as beneficial and no long-term negative consequences have been observed. For example, eliminating p16^Ink4a^ senescent cells with AP20187 attenuated the phenotype of cardiac aging [77]. ABT263, a senolytic drug, could alleviate telomere dysfunction in cardiomyocytes and reduced hypertrophy as well as fibrosis in the hearts [150]. These studies highlight the critical role of cellular senescence for cardiac aging.

## Mechanisms of senotherapeutics

Immunosurveillance decline (such as phagocytosis), chronic low-grade inflammation and SASP secretion are main outcomes of immunosencescence and lead to senescent cell accumulation and proinflammatory phenotypes, greatly contributing to cardiac aging and age-related cardiovascular diseases. Therefore, mitigating inflammation, and restoring/boosting the ability of the immune system as an intrinsic senolytic system could specifically eliminate senescent cells from tissues, serving as a new therapeutic strategy to inhibit the occurrence of age-associated diseases [17]. Poly(I:C), a simulator of viral infection, has been used to stimulate innate immune response to improve senescent cell clearance. And efforts focusing on combining different approches, including anti-inflammatory drugs and immune checkpoint inhibitors, could optimally block inflammation [151].

Senomorphics is mainly targeted to modulate core attributes of senescence, particularly suppressing SASP production and secretion, interfering with the proinflammatory nature of senescent cells and potentially delaying key aspects of aging and aging-associated disease. Modulators of NF-κB signaling, including metformin, apigenin, kaempferol and BAY 11-7082, have been shown to effectively reduce SASP production [152-154]. NLRP3 inflammasome has been shown to be another potential target to suppress SASP for multiple cardiovascular diseases [45, 155]. Colchicine is an inhibitor for tubulin polymerization and the assembly of NLRP3 inflammasome, and could reduce major cardiovascular events (MACE) in patients with chronic stable coronary artery disease [156, 157] as well as those with recent myocardial infarction [158]. In addition, physical exercise could lower inflammation probably by dampening the methylation of the apoptosis-associated protein caspase gene (ASC) and the downstream NLRP3 inflammasome [159]. As an age-related epigenetic regulator, SIRT (Sirtuin) family has been shown to be crucial for macrophage polarization and differentiation. Generally, SIRT1-3 and SIRT6 inhibit the M1 and promote M2 polarization of macrophages, while SIRT4 exerts the opposite effect [160]. As Sirt1/2 activator, resveratrol has been shown to reverse immune aging and inflammation in old mice [161] to improve left ventricular diastolic function, and alleviate cardiac fibrosis [162]. Further research on Sirt-activating compounds/reagents potentially shed more light to treat age-related cardiovascular disease through modulating macrophage senescence.

Monoclonal antibodies have been developed to against some SASP component to alleviate cardiac aging and related diseases in clinical trial [11-13]. For example, canakinumab, a monoclonal antibody against IL-1β, could significantly reduce the risk of MACE in patients with a history of myocardial infarction and elevated levels of high-sensitivity C-reactive protein (hs-CRP) [11, 163]. This CANTOS trial found that canakinumab administration led to a 25% reduction in inflammatory markers, and a 16% reduction in MACE [11].

Nonpharmacological approaches using CAR T or CAR NK cells that target senescent cells are demonstrating promise in pre-clinical and early clinical studies [164]. Meanwhile, immune checkpoint inhibitors, such as inhibitory molecules and monoclonal antibodies against PD1, PD-L1, and cytotoxic T lymphocyte-associated protein 4 (CTLA4), also represent promising strategies for enhancing immunosurveillance [165]. Such approaches depend on the recognition of specific membrane markers on senescent cells. Recent work has foretold a premising potential application of senolytic CAR T cell on attenuating senescence-associated diseases [166]. A CAR-T cell therapy has been developed to deliver modified messenger RNA (mRNA) in T cell-targeted lipid nanoparticles (LNPs) and displayed effect to reduce cardiac fibrosis after injury [167]. However, more efforts are needed to minimize the cardiovascular toxicities associated with this CAR T therapy [168].

Cell transplantation, especially bone marrow transplantation, has been widely used to reestablish immune system in cancer, immune deficiency, and autoimmune disease patients. Although stem cell transplantation aiming to regenerate cardiomyocytes seems to have made no great progress, recent cardiomyocyte-committed iPSC transplantation leaves some hope for heart failure patients [169]. Allow for the critical role of macrophages, especially cardiac macrophages, for cardiac aging, it will be interesting to further investigate whether bone marrow or even differentiated macrophages transplantation works for alleviating cardiac aging.

## Limitations of senotherapeutics

Currently, application of immune-targeted seno-therapeutics for cardiac aging or cardiovascular diseases is just in its incipient stage, probably due to some concerns: As aging is long process, the change for immune cells during this process is hardly synchronous, so it is difficult to identify definite senescent markers for immune cells, even for individual population of immune cells. This heterogeneity of senescent immune cells provides huge hinderances for cell-specific senotherapeutics for cardiac aging. Meanwhile, immune cells are usually distributed ubiquitously in the body, so it is hard to implement senolytics and senomorphics tissue-specifically. Another concern is the immune compromised condition due to lack of specificity of senolytic and senomorphic methods from unintentional killing of immune cells. Additionally, inadvertent activation of immune cells by senotherapeutics, especially senomorphics, may lead to tissue or organ damage.

## Knowledge Gaps and Future Directions

Until recently, most studies on senotherapeutics targeting immunosenescence were based on model animals. Some knowledge gaps have to be filled before its feasible application for alleviating cardiac aging, including (1) The heterogeneity and context-specific roles of senescent immune cells in cardiac tissue; (2) Translational challenges in applying immunosenescence data from murine models to human cardiovascular disease; (3) The current lack of validated biomarkers for immune aging in cardiovascular contexts. For example, growth differentiation factor 11 could reverses age-related cardiac hypertrophy [117], while the role of it for clinical intervention remains to be confirmed. As most immune cells are derived from hematopoietic stem cells (HSC) in bone marrow, so bone marrow transplantation or more precisely, adoptive transfer of induced pluripotent stem cell (iPSC) with commited differentiation to specific immune cells deserves to be explored for cardiac aging. Alternatively, plasmapheresis with young plasma has rejuvenating effects, probably by suppressing the levels of circulating SASP, such as pro-inflammatory and other pro-aging molecules [170]. More investigation on the effect of plasmapheresis for cardiac aging is also interesting, especially to further analyze the precise mediator associated with immunosenescence.

## Conclusion

Despite of the estabilsed role of inflammation in cardiac aging, some key questions remain unsolved: which patient population will benefit from anti-inflammatory therapies and whether simultaneous targeting of several cell types (such as macrophages and T lymphocytes), intracellular pathways (such as the cGAS-STING, NF-κB and inflammasome pathways) or pro-inflammatory effector molecules (such as type I interferons, TNF and IL-1β) might constitute an effective combination strategy for postponing or halting cardiovascular ageing remains to be determined.

## Data Availability

The authors have nothing to report.

## References

[1] Barreda-Ángeles M, Hartmann T (2023). Experiences of Depersonalization/Derealization Among Users of Virtual Reality Applications: A Cross-Sectional Survey. Cyberpsychol Behav Soc Netw, 26:22-27. 10.1089/cyber.2022.0152 36595349

[2] Li Q, Xiao N, Zhang H, Liang G, Lin Y, Qian Z, et al. (2025). Systemic aging and aging-related diseases. Faseb j, 39:e70430. 10.1096/fj.202402479RRR 40022602

[3] García-Domínguez M (2025). Pathological and Inflammatory Consequences of Aging. Biomolecules, 15. 10.3390/biom15030404 PMC11939965 40149940

[4] Sayed N, Huang Y, Nguyen K, Krejciova-Rajaniemi Z, Grawe AP, Gao T, et al. (2021). An inflammatory aging clock (iAge) based on deep learning tracks multimorbidity, immunosenescence, frailty and cardiovascular aging. Nat Aging, 1:598-615. 10.1038/s43587-021-00082-y PMC8654267 34888528

[5] Kasal DA, Sena V, Huguenin GVB, De Lorenzo A, Tibirica E (2025). Microvascular endothelial dysfunction in vascular senescence and disease. Front Cardiovasc Med, 12:1505516. 10.3389/fcvm.2025.1505516 PMC11878104 40041173

[6] Bansilal S, Castellano JM, Fuster V (2015). Global burden of CVD: focus on secondary prevention of cardiovascular disease. Int J Cardiol, 201 Suppl 1:S1-7. 10.1016/S0167-5273(15)31026-3 26747389

[7] Goyani P, Christodoulou R, Vassiliou E (2024). Immunosenescence: Aging and Immune System Decline. Vaccines (Basel), 12. 10.3390/vaccines12121314 PMC11680340 39771976

[8] Pradeu T, Thomma B, Girardin SE, Lemaitre B (2024). The conceptual foundations of innate immunity: Taking stock 30 years later. Immunity, 57:613-631. 10.1016/j.immuni.2024.03.007 38599162

[9] López-Otín C, Blasco MA, Partridge L, Serrano M, Kroemer G (2023). Hallmarks of aging: An expanding universe. Cell, 186:243-278. 10.1016/j.cell.2022.11.001 36599349

[10] Xie S, Xu SC, Deng W, Tang Q (2023). Metabolic landscape in cardiac aging: insights into molecular biology and therapeutic implications. Signal Transduct Target Ther, 8:114. 10.1038/s41392-023-01378-8 PMC10015017 36918543

[11] Ridker PM, Everett BM, Thuren T, MacFadyen JG, Chang WH, Ballantyne C, et al. (2017). Antiinflammatory Therapy with Canakinumab for Atherosclerotic Disease. N Engl J Med, 377:1119-1131. 10.1056/NEJMoa1707914 28845751

[12] Buckley LF, Carbone S, Trankle CR, Canada JM, Erdle CO, Regan JA, et al. (2018). Effect of Interleukin-1 Blockade on Left Ventricular Systolic Performance and Work: A Post Hoc Pooled Analysis of 2 Clinical Trials. J Cardiovasc Pharmacol, 72:68-70. 10.1097/FJC.0000000000000591 PMC6033625 29738374

[13] Buckley LF, Abbate A (2018). Interleukin-1 blockade in cardiovascular diseases: a clinical update. Eur Heart J, 39:2063-2069. 10.1093/eurheartj/ehy128 29584915

[14] Wrona MV, Ghosh R, Coll K, Chun C, Yousefzadeh MJ (2024). The 3 I's of immunity and aging: immunosenescence, inflammaging, and immune resilience. Front Aging, 5:1490302. 10.3389/fragi.2024.1490302 PMC11521913 39478807

[15] Ajoolabady A, Pratico D, Tang D, Zhou S, Franceschi C, Ren J (2024). Immunosenescence and inflammaging: Mechanisms and role in diseases. Ageing Res Rev, 101:102540. 10.1016/j.arr.2024.102540 39395575

[16] Liu Z, Liang Q, Ren Y, Guo C, Ge X, Wang L, et al. (2023). Immunosenescence: molecular mechanisms and diseases. Signal Transduct Target Ther, 8:200. 10.1038/s41392-023-01451-2 PMC10182360 37179335

[17] Mittelbrunn M, Kroemer G (2021). Hallmarks of T cell aging. Nat Immunol, 22:687-698. 10.1038/s41590-021-00927-z 33986548

[18] Salminen A (2021). Immunosuppressive network promotes immunosenescence associated with aging and chronic inflammatory conditions. J Mol Med (Berl), 99:1553-1569. 10.1007/s00109-021-02123-w PMC8384586 34432073

[19] Goronzy JJ, Weyand CM (2019). Mechanisms underlying T cell ageing. Nat Rev Immunol, 19:573-583. 10.1038/s41577-019-0180-1 PMC7584388 31186548

[20] Elyahu Y, Hekselman I, Eizenberg-Magar I, Berner O, Strominger I, Schiller M, et al. (2019). Aging promotes reorganization of the CD4 T cell landscape toward extreme regulatory and effector phenotypes. Sci Adv, 5:eaaw8330. 10.1126/sciadv.aaw8330 PMC6703865 31457092

[21] Mondal AM, Horikawa I, Pine SR, Fujita K, Morgan KM, Vera E, et al. (2013). p53 isoforms regulate aging- and tumor-associated replicative senescence in T lymphocytes. J Clin Invest, 123:5247-5257. 10.1172/JCI70355 PMC3859419 24231352

[22] Thiault N, Darrigues J, Adoue V, Gros M, Binet B, Perals C, et al. (2015). Peripheral regulatory T lymphocytes recirculating to the thymus suppress the development of their precursors. Nat Immunol, 16:628-634. 10.1038/ni.3150 25939024

[23] Quinn KM, Fox A, Harland KL, Russ BE, Li J, Nguyen THO, et al. (2018). Age-Related Decline in Primary CD8(+) T Cell Responses Is Associated with the Development of Senescence in Virtual Memory CD8(+) T Cells. Cell Rep, 23:3512-3524. 10.1016/j.celrep.2018.05.057 29924995

[24] Pereira BI, De Maeyer RPH, Covre LP, Nehar-Belaid D, Lanna A, Ward S, et al. (2020). Sestrins induce natural killer function in senescent-like CD8(+) T cells. Nat Immunol, 21:684-694. 10.1038/s41590-020-0643-3 PMC10249464 32231301

[25] Martyshkina YS, Tereshchenko VP, Bogdanova DA, Rybtsov SA (2023). Reliable Hallmarks and Biomarkers of Senescent Lymphocytes. Int J Mol Sci, 24. 10.3390/ijms242115653 PMC10647376 37958640

[26] Yoshida S, Nakagami H, Hayashi H, Ikeda Y, Sun J, Tenma A, et al. (2020). The CD153 vaccine is a senotherapeutic option for preventing the accumulation of senescent T cells in mice. Nat Commun, 11:2482. 10.1038/s41467-020-16347-w PMC7235045 32424156

[27] Rodrigues LP, Teixeira VR, Alencar-Silva T, Simonassi-Paiva B, Pereira RW, Pogue R, et al. (2021). Hallmarks of aging and immunosenescence: Connecting the dots. Cytokine Growth Factor Rev, 59:9-21. 10.1016/j.cytogfr.2021.01.006 33551332

[28] Pangrazzi L, Weinberger B (2020). T cells, aging and senescence. Exp Gerontol, 134:110887. 10.1016/j.exger.2020.110887 32092501

[29] Pangrazzi L, Reidla J, Carmona Arana JA, Naismith E, Miggitsch C, Meryk A, et al. (2020). CD28 and CD57 define four populations with distinct phenotypic properties within human CD8(+) T cells. Eur J Immunol, 50:363-379. 10.1002/eji.201948362 PMC7079235 31755098

[30] Ratliff M, Alter S, Frasca D, Blomberg BB, Riley RL (2013). In senescence, age-associated B cells secrete TNFα and inhibit survival of B-cell precursors. Aging Cell, 12:303-311. 10.1111/acel.12055 PMC3716274 23410004

[31] Camell CD, Günther P, Lee A, Goldberg EL, Spadaro O, Youm YH, et al. (2019). Aging Induces an Nlrp3 Inflammasome-Dependent Expansion of Adipose B Cells That Impairs Metabolic Homeostasis. Cell Metab, 30:1024-1039.e1026. 10.1016/j.cmet.2019.10.006 PMC6944439 31735593

[32] Gounder SS, Abdullah BJJ, Radzuanb N, Zain F, Sait NBM, Chua C, et al. (2018). Effect of Aging on NK Cell Population and Their Proliferation at Ex Vivo Culture Condition. Anal Cell Pathol (Amst), 2018:7871814. 10.1155/2018/7871814 PMC6098903 30175033

[33] Lee KA, Flores RR, Jang IH, Saathoff A, Robbins PD (2022). Immune Senescence, Immunosenescence and Aging. Front Aging, 3:900028. 10.3389/fragi.2022.900028 PMC9261375 35821850

[34] Hearps AC, Martin GE, Angelovich TA, Cheng WJ, Maisa A, Landay AL, et al. (2012). Aging is associated with chronic innate immune activation and dysregulation of monocyte phenotype and function. Aging Cell, 11:867-875. 10.1111/j.1474-9726.2012.00851.x 22708967

[35] Desdín-Micó G, Soto-Heredero G, Aranda JF, Oller J, Carrasco E, Gabandé-Rodríguez E, et al. (2020). T cells with dysfunctional mitochondria induce multimorbidity and premature senescence. Science, 368:1371-1376. 10.1126/science.aax0860 PMC7616968 32439659

[36] Franceschi C, Garagnani P, Vitale G, Capri M, Salvioli S (2017). Inflammaging and 'Garb-aging'. Trends Endocrinol Metab, 28:199-212. 10.1016/j.tem.2016.09.005 27789101

[37] Di Micco R, Krizhanovsky V, Baker D, d'Adda di Fagagna F (2021). Cellular senescence in ageing: from mechanisms to therapeutic opportunities. Nat Rev Mol Cell Biol, 22:75-95. 10.1038/s41580-020-00314-w PMC8344376 33328614

[38] Davalos AR, Kawahara M, Malhotra GK, Schaum N, Huang J, Ved U, et al. (2013). p53-dependent release of Alarmin HMGB1 is a central mediator of senescent phenotypes. J Cell Biol, 201:613-629. 10.1083/jcb.201206006 PMC3653366 23649808

[39] Wiley CD, Liu S, Limbad C, Zawadzka AM, Beck J, Demaria M, et al. (2019). SILAC Analysis Reveals Increased Secretion of Hemostasis-Related Factors by Senescent Cells. Cell Rep, 28:3329-3337.e3325. 10.1016/j.celrep.2019.08.049 PMC6907691 31553904

[40] Basisty N, Kale A, Jeon OH, Kuehnemann C, Payne T, Rao C, et al. (2020). A proteomic atlas of senescence-associated secretomes for aging biomarker development. PLoS Biol, 18:e3000599. 10.1371/journal.pbio.3000599 PMC6964821 31945054

[41] Khosla S, Farr JN, Tchkonia T, Kirkland JL (2020). The role of cellular senescence in ageing and endocrine disease. Nat Rev Endocrinol, 16:263-275. 10.1038/s41574-020-0335-y PMC7227781 32161396

[42] Childs BG, Baker DJ, Wijshake T, Conover CA, Campisi J, van Deursen JM (2016). Senescent intimal foam cells are deleterious at all stages of atherosclerosis. Science, 354:472-477. 10.1126/science.aaf6659 PMC5112585 27789842

[43] Rodriguez IJ, Lalinde Ruiz N, Llano León M, Martínez Enríquez L, Montilla Velásquez MDP, Ortiz Aguirre JP, et al. (2020). Immunosenescence Study of T Cells: A Systematic Review. Front Immunol, 11:604591. 10.3389/fimmu.2020.604591 PMC7843425 33519813

[44] Tchkonia T, Zhu Y, van Deursen J, Campisi J, Kirkland JL (2013). Cellular senescence and the senescent secretory phenotype: therapeutic opportunities. J Clin Invest, 123:966-972. 10.1172/JCI64098 PMC3582125 23454759

[45] Acosta JC, Banito A, Wuestefeld T, Georgilis A, Janich P, Morton JP, et al. (2013). A complex secretory program orchestrated by the inflammasome controls paracrine senescence. Nat Cell Biol, 15:978-990. 10.1038/ncb2784 PMC3732483 23770676

[46] Li H, Hastings MH, Rhee J, Trager LE, Roh JD, Rosenzweig A (2020). Targeting Age-Related Pathways in Heart Failure. Circ Res, 126:533-551. 10.1161/CIRCRESAHA.119.315889 PMC7041880 32078451

[47] Khavinson V, Linkova N, Dyatlova A, Kantemirova R, Kozlov K (2022). Senescence-Associated Secretory Phenotype of Cardiovascular System Cells and Inflammaging: Perspectives of Peptide Regulation. Cells, 12. 10.3390/cells12010106 PMC9818427 36611900

[48] Nie L, Zhang P, Wang Q, Zhou X, Wang Q (2019). lncRNA-Triggered Macrophage Inflammaging Deteriorates Age-Related Diseases. Mediators Inflamm, 2019:4260309. 10.1155/2019/4260309 PMC6942909 31949425

[49] Xia G, Zhu S, Liu Y, Pan J, Wang X, Shen C, et al. (2024). Transcriptomic profiling and regulatory pathways of cardiac resident macrophages in aging. Cell Mol Life Sci, 81:220. 10.1007/s00018-024-05235-x PMC11102896 38763956

[50] Sendama W (2020). The effect of ageing on the resolution of inflammation. Ageing Res Rev, 57:101000. 10.1016/j.arr.2019.101000 PMC6961112 31862417

[51] De Maeyer RPH, Chambers ES (2021). The impact of ageing on monocytes and macrophages. Immunol Lett, 230:1-10. 10.1016/j.imlet.2020.12.003 33309673

[52] Plowden J, Renshaw-Hoelscher M, Engleman C, Katz J, Sambhara S (2004). Innate immunity in aging: impact on macrophage function. Aging Cell, 3:161-167. 10.1111/j.1474-9728.2004.00102.x 15268749

[53] Markworth JF, Brown LA, Lim E, Castor-Macias JA, Larouche J, Macpherson PCD, et al. (2021). Metabolipidomic profiling reveals an age-related deficiency of skeletal muscle pro-resolving mediators that contributes to maladaptive tissue remodeling. Aging Cell, 20:e13393. 10.1111/acel.13393 PMC8208786 34075679

[54] Halade GV, Lee DH (2022). Inflammation and resolution signaling in cardiac repair and heart failure. EBioMedicine, 79:103992. 10.1016/j.ebiom.2022.103992 PMC9014358 35405389

[55] Huang W, Hickson LJ, Eirin A, Kirkland JL, Lerman LO (2022). Cellular senescence: the good, the bad and the unknown. Nat Rev Nephrol, 18:611-627. 10.1038/s41581-022-00601-z PMC9362342 35922662

[56] Takahashi A, Loo TM, Okada R, Kamachi F, Watanabe Y, Wakita M, et al. (2018). Downregulation of cytoplasmic DNases is implicated in cytoplasmic DNA accumulation and SASP in senescent cells. Nat Commun, 9:1249. 10.1038/s41467-018-03555-8 PMC5871854 29593264

[57] Hamann L, Ruiz-Moreno JS, Szwed M, Mossakowska M, Lundvall L, Schumann RR, et al. (2019). STING SNP R293Q Is Associated with a Decreased Risk of Aging-Related Diseases. Gerontology, 65:145-154. 10.1159/000492972 30368497

[58] Jeremiah N, Neven B, Gentili M, Callebaut I, Maschalidi S, Stolzenberg MC, et al. (2014). Inherited STING-activating mutation underlies a familial inflammatory syndrome with lupus-like manifestations. J Clin Invest, 124:5516-5520. 10.1172/JCI79100 PMC4348945 25401470

[59] Herranz N, Gallage S, Mellone M, Wuestefeld T, Klotz S, Hanley CJ, et al. (2015). mTOR regulates MAPKAPK2 translation to control the senescence-associated secretory phenotype. Nat Cell Biol, 17:1205-1217. 10.1038/ncb3225 PMC4589897 26280535

[60] Ajoolabady A, Aslkhodapasandhokmabad H, Aghanejad A, Zhang Y, Ren J (2020). Mitophagy Receptors and Mediators: Therapeutic Targets in the Management of Cardiovascular Ageing. Ageing Res Rev, 62:101129. 10.1016/j.arr.2020.101129 32711157

[61] Cordero MD, Williams MR, Ryffel B (2018). AMP-Activated Protein Kinase Regulation of the NLRP3 Inflammasome during Aging. Trends Endocrinol Metab, 29:8-17. 10.1016/j.tem.2017.10.009 29150317

[62] Pellegrini C, Martelli A, Antonioli L, Fornai M, Blandizzi C, Calderone V (2021). NLRP3 inflammasome in cardiovascular diseases: Pathophysiological and pharmacological implications. Med Res Rev, 41:1890-1926. 10.1002/med.21781 33460162

[63] Marín-Aguilar F, Lechuga-Vieco AV, Alcocer-Gómez E, Castejón-Vega B, Lucas J, Garrido C, et al. (2020). NLRP3 inflammasome suppression improves longevity and prevents cardiac aging in male mice. Aging Cell, 19:e13050. 10.1111/acel.13050 PMC6974709 31625260

[64] Toldo S, Mezzaroma E, Buckley LF, Potere N, Di Nisio M, Biondi-Zoccai G, et al. (2022). Targeting the NLRP3 inflammasome in cardiovascular diseases. Pharmacol Ther, 236:108053. 10.1016/j.pharmthera.2021.108053 PMC9187780 34906598

[65] Li R, Lu K, Wang Y, Chen M, Zhang F, Shen H, et al. (2017). Triptolide attenuates pressure overload-induced myocardial remodeling in mice via the inhibition of NLRP3 inflammasome expression. Biochem Biophys Res Commun, 485:69-75. 10.1016/j.bbrc.2017.02.021 28202417

[66] Sano S, Oshima K, Wang Y, MacLauchlan S, Katanasaka Y, Sano M, et al. (2018). Tet2-Mediated Clonal Hematopoiesis Accelerates Heart Failure Through a Mechanism Involving the IL-1β/NLRP3 Inflammasome. J Am Coll Cardiol, 71:875-886. 10.1016/j.jacc.2017.12.037 PMC5828038 29471939

[67] Li X, Geng J, Zhao J, Ni Q, Zhao C, Zheng Y, et al. (2019). Trimethylamine N-Oxide Exacerbates Cardiac Fibrosis via Activating the NLRP3 Inflammasome. Front Physiol, 10:866. 10.3389/fphys.2019.00866 PMC6634262 31354519

[68] Butts B, Gary RA, Dunbar SB, Butler J (2015). The Importance of NLRP3 Inflammasome in Heart Failure. J Card Fail, 21:586-593. 10.1016/j.cardfail.2015.04.014 PMC4516025 25982825

[69] Björkegren JLM, Lusis AJ (2022). Atherosclerosis: Recent developments. Cell, 185:1630-1645. 10.1016/j.cell.2022.04.004 PMC9119695 35504280

[70] Lv C, Alimu X, Xiao X, Wang F, Wang J, Wang S, et al. (2025). Somatic NAP1L1 p.D349E promotes cardiac hypertrophy through cGAS-STING-IFN signaling. Nat Commun, 16:3140. 10.1038/s41467-025-58453-7 PMC11961713 40169585

[71] Lu L, Liu X, Gong Y, Zhou X, Chen A, Li M, et al. (2025). STING Facilitates Vascular Calcification via p-STAT1/NLRP3 Signal. Faseb j, 39:e70530. 10.1096/fj.202403334R 40193045

[72] Marin I, Boix O, Garcia-Garijo A, Sirois I, Caballe A, Zarzuela E, et al. (2023). Cellular Senescence Is Immunogenic and Promotes Antitumor Immunity. Cancer Discov, 13:410-431. 10.1158/2159-8290.CD-22-0523 PMC7614152 36302218

[73] Moskalev A, Stambler I, Caruso C (2020). Innate and Adaptive Immunity in Aging and Longevity: The Foundation of Resilience. Aging Dis, 11:1363-1373. 10.14336/AD.2020.0603 PMC7673842 33269094

[74] Yousefzadeh MJ, Flores RR, Zhu Y, Schmiechen ZC, Brooks RW, Trussoni CE, et al. (2021). An aged immune system drives senescence and ageing of solid organs. Nature, 594:100-105. 10.1038/s41586-021-03547-7 PMC8684299 33981041

[75] Yang HS, Zhang C, Carlyle BC, Zhen SY, Trombetta BA, Schultz AP, et al. (2022). Plasma IL-12/IFN-γ axis predicts cognitive trajectories in cognitively unimpaired older adults. Alzheimers Dement, 18:645-653. 10.1002/alz.12399 PMC8695625 34160128

[76] Holodick NE, Vizconde T, Hopkins TJ, Rothstein TL (2016). Age-Related Decline in Natural IgM Function: Diversification and Selection of the B-1a Cell Pool with Age. J Immunol, 196:4348-4357. 10.4049/jimmunol.1600073 PMC4874569 27183643

[77] Kendrick ME (2006). Cholesterol and risk of stroke: the Japanese comparison. Bmj, 333:148-149. 10.1136/bmj.333.7559.148-b PMC1502244 16840489

[78] Ji H, Kwan AC, Chen MT, Ouyang D, Ebinger JE, Bell SP, et al. (2022). Sex Differences in Myocardial and Vascular Aging. Circ Res, 130:566-577. 10.1161/CIRCRESAHA.121.319902 PMC8863105 35175845

[79] Doenst T, Nguyen TD, Abel ED (2013). Cardiac metabolism in heart failure: implications beyond ATP production. Circ Res, 113:709-724. 10.1161/CIRCRESAHA.113.300376 PMC3896379 23989714

[80] Kang J, Rhee J, Wang C, Yang Y, Li G, Li H (2024). Unlocking the dark matter: noncoding RNAs and RNA modifications in cardiac aging. Am J Physiol Heart Circ Physiol, 326:H832-h844. 10.1152/ajpheart.00532.2023 PMC11221808 38305752

[81] Hoshino A, Mita Y, Okawa Y, Ariyoshi M, Iwai-Kanai E, Ueyama T, et al. (2013). Cytosolic p53 inhibits Parkin-mediated mitophagy and promotes mitochondrial dysfunction in the mouse heart. Nat Commun, 4:2308. 10.1038/ncomms3308 23917356

[82] Lopaschuk GD, Karwi QG, Tian R, Wende AR, Abel ED (2021). Cardiac Energy Metabolism in Heart Failure. Circ Res, 128:1487-1513. 10.1161/CIRCRESAHA.121.318241 PMC8136750 33983836

[83] Roh J, Rhee J, Chaudhari V, Rosenzweig A (2016). The Role of Exercise in Cardiac Aging: From Physiology to Molecular Mechanisms. Circ Res, 118:279-295. 10.1161/CIRCRESAHA.115.305250 PMC4914047 26838314

[84] Rossiello F, Aguado J, Sepe S, Iannelli F, Nguyen Q, Pitchiaya S, et al. (2017). DNA damage response inhibition at dysfunctional telomeres by modulation of telomeric DNA damage response RNAs. Nat Commun, 8:13980. 10.1038/ncomms13980 PMC5473644 28239143

[85] Li J, Xin Y, Wang Z, Li J, Li W, Li H (2023). The role of cardiac resident macrophage in cardiac aging. Aging Cell, 22:e14008. 10.1111/acel.14008 PMC10726886 37817547

[86] Abdellatif M, Rainer PP, Sedej S, Kroemer G (2023). Hallmarks of cardiovascular ageing. Nat Rev Cardiol, 20:754-777. 10.1038/s41569-023-00881-3 37193857

[87] Liao Y, Zhu L (2024). At the heart of inflammation: Unravelling cardiac resident macrophage biology. J Cell Mol Med, 28:e70050. 10.1111/jcmm.70050 PMC11369210 39223947

[88] Holt M, Lin J, Cicka M, Wong A, Epelman S, Lavine KJ (2024). Dissecting and Visualizing the Functional Diversity of Cardiac Macrophages. Circ Res, 134:1791-1807. 10.1161/CIRCRESAHA.124.323817 PMC12772442 38843293

[89] Cai W, Lian L, Li A, Zhang Q, Li M, Zhang J, et al. (2024). Cardiac resident macrophages: The core of cardiac immune homeostasis. Cell Signal, 119:111169. 10.1016/j.cellsig.2024.111169 38599440

[90] Kopecky BJ, Lavine KJ (2024). Cardiac macrophage metabolism in health and disease. Trends Endocrinol Metab, 35:249-262. 10.1016/j.tem.2023.10.011 PMC10949041 37993313

[91] Revelo XS, Parthiban P, Chen C, Barrow F, Fredrickson G, Wang H, et al. (2021). Cardiac Resident Macrophages Prevent Fibrosis and Stimulate Angiogenesis. Circ Res, 129:1086-1101. 10.1161/CIRCRESAHA.121.319737 PMC8638822 34645281

[92] Bajpai G, Bredemeyer A, Li W, Zaitsev K, Koenig AL, Lokshina I, et al. (2019). Tissue Resident CCR2- and CCR2+ Cardiac Macrophages Differentially Orchestrate Monocyte Recruitment and Fate Specification Following Myocardial Injury. Circ Res, 124:263-278. 10.1161/CIRCRESAHA.118.314028 PMC6626616 30582448

[93] Nicolás-Ávila JA, Lechuga-Vieco AV, Esteban-Martínez L, Sánchez-Díaz M, Díaz-García E, Santiago DJ, et al. (2020). A Network of Macrophages Supports Mitochondrial Homeostasis in the Heart. Cell, 183:94-109.e123. 10.1016/j.cell.2020.08.031 32937105

[94] Wang Y, Sano S, Yura Y, Ke Z, Sano M, Oshima K, et al. (2020). Tet2-mediated clonal hematopoiesis in nonconditioned mice accelerates age-associated cardiac dysfunction. JCI Insight, 5. 10.1172/jci.insight.135204 PMC7213793 32154790

[95] Varricchi G, Bencivenga L, Poto R, Pecoraro A, Shamji MH, Rengo G (2020). The emerging role of T follicular helper (T(FH)) cells in aging: Influence on the immune frailty. Ageing Res Rev, 61:101071. 10.1016/j.arr.2020.101071 32344191

[96] Pinto AR, Godwin JW, Chandran A, Hersey L, Ilinykh A, Debuque R, et al. (2014). Age-related changes in tissue macrophages precede cardiac functional impairment. Aging (Albany NY), 6:399-413. 10.18632/aging.100669 PMC4069267 24861132

[97] Ferrucci L, Fabbri E (2018). Inflammageing: chronic inflammation in ageing, cardiovascular disease, and frailty. Nat Rev Cardiol, 15:505-522. 10.1038/s41569-018-0064-2 PMC6146930 30065258

[98] Barbé-Tuana F, Funchal G, Schmitz CRR, Maurmann RM, Bauer ME (2020). The interplay between immunosenescence and age-related diseases. Semin Immunopathol, 42:545-557. 10.1007/s00281-020-00806-z PMC7398288 32747977

[99] Shaw AC, Goldstein DR, Montgomery RR (2013). Age-dependent dysregulation of innate immunity. Nat Rev Immunol, 13:875-887. 10.1038/nri3547 PMC4096436 24157572

[100] Kane AE, Bisset ES, Heinze-Milne S, Keller KM, Grandy SA, Howlett SE (2021). Maladaptive Changes Associated With Cardiac Aging Are Sex-Specific and Graded by Frailty and Inflammation in C57BL/6 Mice. J Gerontol A Biol Sci Med Sci, 76:233-243. 10.1093/gerona/glaa212 PMC7812442 32857156

[101] Dosch M, Zindel J, Jebbawi F, Melin N, Sanchez-Taltavull D, Stroka D, et al. (2019). Connexin-43-dependent ATP release mediates macrophage activation during sepsis. Elife, 8. 10.7554/eLife.42670 PMC6415938 30735126

[102] Hulsmans M, Clauss S, Xiao L, Aguirre AD, King KR, Hanley A, et al. (2017). Macrophages Facilitate Electrical Conduction in the Heart. Cell, 169:510-522.e520. 10.1016/j.cell.2017.03.050 PMC5474950 28431249

[103] Simon-Chica A, Fernández MC, Wülfers EM, Lother A, Hilgendorf I, Seemann G, et al. (2022). Novel insights into the electrophysiology of murine cardiac macrophages: relevance of voltage-gated potassium channels. Cardiovasc Res, 118:798-813. 10.1093/cvr/cvab126 PMC8859634 33823533

[104] Sugita J, Fujiu K, Nakayama Y, Matsubara T, Matsuda J, Oshima T, et al. (2021). Cardiac macrophages prevent sudden death during heart stress. Nat Commun, 12:1910. 10.1038/s41467-021-22178-0 PMC7997915 33771995

[105] Ding C, Tang G, Sun Y, Fu X, Tian Y, Zhan J, et al. (2025). A functional cardiac patch promotes cardiac repair by modulating the CCR2(-) cardiac-resident macrophage niche and their cell crosstalk. Cell Rep Med, 6:101932. 10.1016/j.xcrm.2025.101932 PMC11866506 39879993

[106] Ezzo M, Spindler K, Wang JB, Lee D, Pecoraro G, Cowen J, et al. (2024). Acute contact with profibrotic macrophages mechanically activates fibroblasts via αvβ3 integrin-mediated engagement of Piezo1. Sci Adv, 10:eadp4726. 10.1126/sciadv.adp4726 PMC11498225 39441936

[107] He A, Shi GP (2013). Mast cell chymase and tryptase as targets for cardiovascular and metabolic diseases. Curr Pharm Des, 19:1114-1125. 10.2174/1381612811319060012 PMC3921624 23016684

[108] Ngkelo A, Richart A, Kirk JA, Bonnin P, Vilar J, Lemitre M, et al. (2016). Mast cells regulate myofilament calcium sensitization and heart function after myocardial infarction. J Exp Med, 213:1353-1374. 10.1084/jem.20160081 PMC4925026 27353089

[109] Ashour D, Rebs S, Arampatzi P, Saliba AE, Dudek J, Schulz R, et al. (2023). An interferon gamma response signature links myocardial aging and immunosenescence. Cardiovasc Res, 119:2458-2468. 10.1093/cvr/cvad068 PMC10651211 37141306

[110] Ramos GC, van den Berg A, Nunes-Silva V, Weirather J, Peters L, Burkard M, et al. (2017). Myocardial aging as a T-cell-mediated phenomenon. Proc Natl Acad Sci U S A, 114:E2420-e2429. 10.1073/pnas.1621047114 PMC5373357 28255084

[111] Delgobo M, Heinrichs M, Hapke N, Ashour D, Appel M, Srivastava M, et al. (2021). Terminally Differentiated CD4(+) T Cells Promote Myocardial Inflammaging. Front Immunol, 12:584538. 10.3389/fimmu.2021.584538 PMC7935504 33679735

[112] Dolejsi T, Delgobo M, Schuetz T, Tortola L, Heinze KG, Hofmann U, et al. (2022). Adult T-cells impair neonatal cardiac regeneration. Eur Heart J, 43:2698-2709. 10.1093/eurheartj/ehac153 PMC9300388 35417553

[113] Haghikia A, Kaya Z, Schwab J, Westenfeld R, Ehlermann P, Bachelier K, et al. (2015). Evidence of autoantibodies against cardiac troponin I and sarcomeric myosin in peripartum cardiomyopathy. Basic Res Cardiol, 110:60. 10.1007/s00395-015-0517-2 26519371

[114] Del Pinto R, Ferri C (2018). Inflammation-Accelerated Senescence and the Cardiovascular System: Mechanisms and Perspectives. Int J Mol Sci, 19. 10.3390/ijms19123701 PMC6321367 30469478

[115] Anderson R, Lagnado A, Maggiorani D, Walaszczyk A, Dookun E, Chapman J, et al. (2019). Length-independent telomere damage drives post-mitotic cardiomyocyte senescence. Embo j, 38. 10.15252/embj.2018100492 PMC6396144 30737259

[116] Roh JD, Hobson R, Chaudhari V, Quintero P, Yeri A, Benson M, et al. (2019). Activin type II receptor signaling in cardiac aging and heart failure. Sci Transl Med, 11. 10.1126/scitranslmed.aau8680 PMC7124007 30842316

[117] Loffredo FS, Steinhauser ML, Jay SM, Gannon J, Pancoast JR, Yalamanchi P, et al. (2013). Growth differentiation factor 11 is a circulating factor that reverses age-related cardiac hypertrophy. Cell, 153:828-839. 10.1016/j.cell.2013.04.015 PMC3677132 23663781

[118] Markousis-Mavrogenis G, Habib M, Huck DM, André F, Steen H, Mukherjee M, et al. (2025). Emerging Imaging Techniques for Atherosclerosis in Systemic Immune-Mediated Inflammatory Conditions. Arterioscler Thromb Vasc Biol, 45:11-22. 10.1161/ATVBAHA.124.321202 PMC12055239 39540283

[119] Holdt LM, Sass K, Gäbel G, Bergert H, Thiery J, Teupser D (2011). Expression of Chr9p21 genes CDKN2B (p15(INK4b)), CDKN2A (p16(INK4a), p14(ARF)) and MTAP in human atherosclerotic plaque. Atherosclerosis, 214:264-270. 10.1016/j.atherosclerosis.2010.06.029 20637465

[120] Minamino T, Miyauchi H, Yoshida T, Ishida Y, Yoshida H, Komuro I (2002). Endothelial cell senescence in human atherosclerosis: role of telomere in endothelial dysfunction. Circulation, 105:1541-1544. 10.1161/01.cir.0000013836.85741.17 11927518

[121] Morgan RG, Ives SJ, Lesniewski LA, Cawthon RM, Andtbacka RH, Noyes RD, et al. (2013). Age-related telomere uncapping is associated with cellular senescence and inflammation independent of telomere shortening in human arteries. Am J Physiol Heart Circ Physiol, 305:H251-258. 10.1152/ajpheart.00197.2013 PMC3726958 23666675

[122] Matthews C, Gorenne I, Scott S, Figg N, Kirkpatrick P, Ritchie A, et al. (2006). Vascular smooth muscle cells undergo telomere-based senescence in human atherosclerosis: effects of telomerase and oxidative stress. Circ Res, 99:156-164. 10.1161/01.RES.0000233315.38086.bc 16794190

[123] Hu C, Zhang X, Teng T, Ma ZG, Tang QZ (2022). Cellular Senescence in Cardiovascular Diseases: A Systematic Review. Aging Dis, 13:103-128. 10.14336/AD.2021.0927 PMC8782554 35111365

[124] Stojanović SD, Fiedler J, Bauersachs J, Thum T, Sedding DG (2020). Senescence-induced inflammation: an important player and key therapeutic target in atherosclerosis. Eur Heart J, 41:2983-2996. 10.1093/eurheartj/ehz919 PMC7453834 31898722

[125] Bloom SI, Islam MT, Lesniewski LA, Donato AJ (2023). Mechanisms and consequences of endothelial cell senescence. Nat Rev Cardiol, 20:38-51. 10.1038/s41569-022-00739-0 PMC10026597 35853997

[126] Barcena ML, Aslam M, Pozdniakova S, Norman K, Ladilov Y (2022). Cardiovascular Inflammaging: Mechanisms and Translational Aspects. Cells, 11. 10.3390/cells11061010 PMC8946971 35326461

[127] Laina A, Stellos K, Stamatelopoulos K (2018). Vascular ageing: Underlying mechanisms and clinical implications. Exp Gerontol, 109:16-30. 10.1016/j.exger.2017.06.007 28624356

[128] Lakatta EG (2003). Arterial and cardiac aging: major shareholders in cardiovascular disease enterprises: Part III: cellular and molecular clues to heart and arterial aging. Circulation, 107:490-497. 10.1161/01.cir.0000048894.99865.02 12551876

[129] Ajoolabady A, Bi Y, McClements DJ, Lip GYH, Richardson DR, Reiter RJ, et al. (2022). Melatonin-based therapeutics for atherosclerotic lesions and beyond: Focusing on macrophage mitophagy. Pharmacol Res, 176:106072. 10.1016/j.phrs.2022.106072 35007709

[130] Duan J, Dong W, Wang G, Xiu W, Pu G, Xu J, et al. (2023). Senescence-associated 13-HODE production promotes age-related liver steatosis by directly inhibiting catalase activity. Nat Commun, 14:8151. 10.1038/s41467-023-44026-z PMC10710422 38071367

[131] Abbas A, Aukrust P, Russell D, Krohg-Sørensen K, Almås T, Bundgaard D, et al. (2014). Matrix metalloproteinase 7 is associated with symptomatic lesions and adverse events in patients with carotid atherosclerosis. PLoS One, 9:e84935. 10.1371/journal.pone.0084935 PMC3882279 24400123

[132] Olejarz W, Łacheta D, Kubiak-Tomaszewska G (2020). Matrix Metalloproteinases as Biomarkers of Atherosclerotic Plaque Instability. Int J Mol Sci, 21. 10.3390/ijms21113946 PMC7313469 32486345

[133] Wågsäter D, Zhu C, Björkegren J, Skogsberg J, Eriksson P (2011). MMP-2 and MMP-9 are prominent matrix metalloproteinases during atherosclerosis development in the Ldlr(-/-)Apob(100/100) mouse. Int J Mol Med, 28:247-253. 10.3892/ijmm.2011.693 21567073

[134] Barcena ML, Pozdniakova S, Haritonow N, Breiter P, Kühl AA, Milting H, et al. (2020). Dilated cardiomyopathy impairs mitochondrial biogenesis and promotes inflammation in an age- and sex-dependent manner. Aging (Albany NY), 12:24117-24133. 10.18632/aging.202283 PMC7762497 33303703

[135] Díez-Villanueva P, Jiménez-Méndez C, Alfonso F (2021). Heart failure in the elderly. J Geriatr Cardiol, 18:219-232. 10.11909/j.issn.1671-5411.2021.03.009 PMC8047189 33907552

[136] Ebong IA, Gill A, Appiah D, Mauricio R (2025). Sex differences in the presentation, pathophysiology, and prognosis of heart failure with preserved ejection fraction. Heart Fail Rev. 10.1007/s10741-025-10528-9 40445492

[137] Sanders-van Wijk S, van Empel V, Davarzani N, Maeder MT, Handschin R, Pfisterer ME, et al. (2015). Circulating biomarkers of distinct pathophysiological pathways in heart failure with preserved vs. reduced left ventricular ejection fraction. Eur J Heart Fail, 17:1006-1014. 10.1002/ejhf.414 26472682

[138] Pugliese NR, Pellicori P, Filidei F, De Biase N, Maffia P, Guzik TJ, et al. (2023). Inflammatory pathways in heart failure with preserved left ventricular ejection fraction: implications for future interventions. Cardiovasc Res, 118:3536-3555. 10.1093/cvr/cvac133 PMC9897694 36004819

[139] Maier HJ, Schips TG, Wietelmann A, Krüger M, Brunner C, Sauter M, et al. (2012). Cardiomyocyte-specific IκB kinase (IKK)/NF-κB activation induces reversible inflammatory cardiomyopathy and heart failure. Proc Natl Acad Sci U S A, 109:11794-11799. 10.1073/pnas.1116584109 PMC3406816 22753500

[140] Hasegawa Y, Saito T, Ogihara T, Ishigaki Y, Yamada T, Imai J, et al. (2012). Blockade of the nuclear factor-κB pathway in the endothelium prevents insulin resistance and prolongs life spans. Circulation, 125:1122-1133. 10.1161/CIRCULATIONAHA.111.054346 22302838

[141] Kubota T, McTiernan CF, Frye CS, Slawson SE, Lemster BH, Koretsky AP, et al. (1997). Dilated cardiomyopathy in transgenic mice with cardiac-specific overexpression of tumor necrosis factor-alpha. Circ Res, 81:627-635. 10.1161/01.res.81.4.627 9314845

[142] Everett BM, Cornel JH, Lainscak M, Anker SD, Abbate A, Thuren T, et al. (2019). Anti-Inflammatory Therapy With Canakinumab for the Prevention of Hospitalization for Heart Failure. Circulation, 139:1289-1299. 10.1161/CIRCULATIONAHA.118.038010 30586730

[143] Chen R, Xie Y, Chang Z, Hu W, Han Z (2024). Integration of single-cell sequencing with machine learning and Mendelian randomization analysis identifies the NAP1L1 gene as a predictive biomarker for Alzheimer's disease. Front Aging Neurosci, 16:1406160. 10.3389/fnagi.2024.1406160 PMC11233722 38988327

[144] Liao YH, Xia N, Zhou SF, Tang TT, Yan XX, Lv BJ, et al. (2012). Interleukin-17A contributes to myocardial ischemia/reperfusion injury by regulating cardiomyocyte apoptosis and neutrophil infiltration. J Am Coll Cardiol, 59:420-429. 10.1016/j.jacc.2011.10.863 PMC3262985 22261166

[145] Du S, Li Z, Xie X, Xu C, Shen X, Wang N, et al. (2020). IL-17 stimulates the expression of CCL2 in cardiac myocytes via Act1/TRAF6/p38MAPK-dependent AP-1 activation. Scand J Immunol, 91:e12840. 10.1111/sji.12840 31630418

[146] Kallikourdis M, Martini E, Carullo P, Sardi C, Roselli G, Greco CM, et al. (2017). T cell costimulation blockade blunts pressure overload-induced heart failure. Nat Commun, 8:14680. 10.1038/ncomms14680 PMC5343521 28262700

[147] Yin Y, Han X, Shi Q, Zhao Y, He Y (2012). Adoptive transfer of CD4+CD25+ regulatory T cells for prevention and treatment of spontaneous abortion. Eur J Obstet Gynecol Reprod Biol, 161:177-181. 10.1016/j.ejogrb.2011.12.023 22261465

[148] de Magalhães JP (2024). Cellular senescence in normal physiology. Science, 384:1300-1301. 10.1126/science.adj7050 38900869

[149] Childs BG, Gluscevic M, Baker DJ, Laberge RM, Marquess D, Dananberg J, et al. (2017). Senescent cells: an emerging target for diseases of ageing. Nat Rev Drug Discov, 16:718-735. 10.1038/nrd.2017.116 PMC5942225 28729727

[150] Yu M, Shan X, Bai Y, Qin Y, Zhao X (2019). Percutaneous closure of a large atrial septal defect presenting with acute severe hemolysis. Clin Res Cardiol, 108:828-830. 10.1007/s00392-019-01431-z 30737529

[151] Chambers ES, Akbar AN (2020). Can blocking inflammation enhance immunity during aging^+^ J Allergy Clin Immunol, 145:1323-1331. 10.1016/j.jaci.2020.03.016 32386656

[152] Kim YS, Kim JS, Kwon JS, Jeong MH, Cho JG, Park JC, et al. (2010). BAY 11-7082, a nuclear factor-κB inhibitor, reduces inflammation and apoptosis in a rat cardiac ischemia-reperfusion injury model. Int Heart J, 51:348-353. 10.1536/ihj.51.348 20966608

[153] Cook M, Lin H, Mishra SK, Wang GY (2022). BAY 11-7082 inhibits the secretion of interleukin-6 by senescent human microglia. Biochem Biophys Res Commun, 617:30-35. 10.1016/j.bbrc.2022.05.090 PMC9540971 35671608

[154] Lopes-Paciencia S, Saint-Germain E, Rowell MC, Ruiz AF, Kalegari P, Ferbeyre G (2019). The senescence-associated secretory phenotype and its regulation. Cytokine, 117:15-22. 10.1016/j.cyto.2019.01.013 30776684

[155] Rea IM, Gibson DS, McGilligan V, McNerlan SE, Alexander HD, Ross OA (2018). Age and Age-Related Diseases: Role of Inflammation Triggers and Cytokines. Front Immunol, 9:586. 10.3389/fimmu.2018.00586 PMC5900450 29686666

[156] Nidorf SM, Fiolet ATL, Mosterd A, Eikelboom JW, Schut A, Opstal TSJ, et al. (2020). Colchicine in Patients with Chronic Coronary Disease. N Engl J Med, 383:1838-1847. 10.1056/NEJMoa2021372 32865380

[157] Nidorf SM, Eikelboom JW, Budgeon CA, Thompson PL (2013). Low-dose colchicine for secondary prevention of cardiovascular disease. J Am Coll Cardiol, 61:404-410. 10.1016/j.jacc.2012.10.027 23265346

[158] Tardif JC, Kouz S, Waters DD, Bertrand OF, Diaz R, Maggioni AP, et al. (2019). Efficacy and Safety of Low-Dose Colchicine after Myocardial Infarction. N Engl J Med, 381:2497-2505. 10.1056/NEJMoa1912388 31733140

[159] Butts B, Butler J, Dunbar SB, Corwin E, Gary RA (2018). Effects of Exercise on ASC Methylation and IL-1 Cytokines in Heart Failure. Med Sci Sports Exerc, 50:1757-1766. 10.1249/MSS.0000000000001641 PMC6095733 29683921

[160] Ding YN, Wang HY, Chen XF, Tang X, Chen HZ (2025). Roles of Sirtuins in Cardiovascular Diseases: Mechanisms and Therapeutics. Circ Res, 136:524-550. 10.1161/CIRCRESAHA.124.325440 40014680

[161] Wong YT, Gruber J, Jenner AM, Tay FE, Ruan R (2011). Chronic resveratrol intake reverses pro-inflammatory cytokine profile and oxidative DNA damage in ageing hybrid mice. Age (Dordr), 33:229-246. 10.1007/s11357-010-9174-4 PMC3168607 20730501

[162] Zheng X, Hai J, Yang Y, Zhang C, Ma X, Kong B, et al. (2023). Effects of resveratrol supplementation on cardiac remodeling in hypertensive patients: a randomized controlled clinical trial. Hypertens Res, 46:1493-1503. 10.1038/s41440-023-01231-z 36854725

[163] Tall AR, Bornfeldt KE (2023). Inflammasomes and Atherosclerosis: a Mixed Picture. Circ Res, 132:1505-1520. 10.1161/CIRCRESAHA.123.321637 PMC10213995 37228237

[164] Feucht J, Abou-El-Enein M (2020). Senolytic CAR T Cells in Solid Tumors and Age-Related Pathologies. Mol Ther, 28:2108-2110. 10.1016/j.ymthe.2020.08.011 PMC7545000 32841587

[165] Sharma P, Goswami S, Raychaudhuri D, Siddiqui BA, Singh P, Nagarajan A, et al. (2023). Immune checkpoint therapy-current perspectives and future directions. Cell, 186:1652-1669. 10.1016/j.cell.2023.03.006 37059068

[166] Amor C, Feucht J, Leibold J, Ho YJ, Zhu C, Alonso-Curbelo D, et al. (2020). Senolytic CAR T cells reverse senescence-associated pathologies. Nature, 583:127-132. 10.1038/s41586-020-2403-9 PMC7583560 32555459

[167] Rurik JG, Tombácz I, Yadegari A, Méndez Fernández PO, Shewale SV, Li L, et al. (2022). CAR T cells produced in vivo to treat cardiac injury. Science, 375:91-96. 10.1126/science.abm0594 PMC9983611 34990237

[168] Liu JH, Liu KY, Zhao X, Zhou X, Jiang Y (2025). Cardiovascular toxicities associated with chimeric antigen receptor T-cell therapy. Front Pharmacol, 16:1578157. 10.3389/fphar.2025.1578157 PMC12094984 40406483

[169] Kishino Y, Tohyama S, Morita Y, Soma Y, Tani H, Okada M, et al. (2023). Cardiac Regenerative Therapy Using Human Pluripotent Stem Cells for Heart Failure: A State-of-the-Art Review. J Card Fail, 29:503-513. 10.1016/j.cardfail.2022.10.433 37059512

[170] Borsky P, Holmannova D, Parova H, Horvath S, Sramek P, Brooke RT, et al. (2025). Human clinical trial of plasmapheresis effects on biomarkers of aging (efficacy and safety trial). Sci Rep, 15:21059. 10.1038/s41598-025-05396-0 PMC12218284 40592961

[171] Zhang X, Zhao Q, Wang T, Long Q, Sun Y, Jiao L, et al. (2023). DNA damage response, a double-edged sword for vascular aging. Ageing Res Rev, 92:102137. 10.1016/j.arr.2023.102137 38007046

[172] An T, Guo M, Wang Z, Liu K (2025). Tissue-Resident Macrophages in Cardiovascular Diseases: Heterogeneity and Therapeutic Potential. Int J Mol Sci, 26. 10.3390/ijms26104524 PMC12111180 40429668

[173] Sen I, Trzaskalski NA, Hsiao YT, Liu PP, Shimizu I, Derumeaux GA (2025). Aging at the Crossroads of Organ Interactions: Implications for the Heart. Circ Res, 136:1286-1305. 10.1161/CIRCRESAHA.125.325637 40403108

[174] Warren CM, Halas M, Goldspink PH, Feng HZ, Herren AW, Wolska BM, et al. (2022). Truncation of the N-terminus of cardiac troponin I initiates adaptive remodeling of the myocardial proteosome via phosphorylation of mechano-sensitive signaling pathways. Mol Cell Biochem, 477:1803-1815. 10.1007/s11010-022-04414-3 PMC10329822 35316461

[175] Nguyen TT, Corvera S (2024). Adipose tissue as a linchpin of organismal ageing. Nat Metab, 6:793-807. 10.1038/s42255-024-01046-3 PMC11238912 38783156

[176] Smit V, de Mol J, Schaftenaar FH, Depuydt MAC, Postel RJ, Smeets D, et al. (2023). Single-cell profiling reveals age-associated immunity in atherosclerosis. Cardiovasc Res, 119:2508-2521. 10.1093/cvr/cvad099 PMC10676459 37390467

[177] Sebastian RS, Hoy MK, Goldman JD, Moshfegh AJ.2010. Snack Consumption by U.S. Adults: What We Eat in America, NHANES 2017 - March 2020. In FSRG Dietary Data Briefs. Beltsville (MD): United States Department of Agriculture (USDA). 38768292

[178] Kolluru GK, Shen X, Kevil CG (2020). Reactive Sulfur Species: A New Redox Player in Cardiovascular Pathophysiology. Arterioscler Thromb Vasc Biol, 40:874-884. 10.1161/ATVBAHA.120.314084 PMC7098439 32131614

[179] Weidinger A, Kozlov AV (2015). Biological Activities of Reactive Oxygen and Nitrogen Species: Oxidative Stress versus Signal Transduction. Biomolecules, 5:472-484. 10.3390/biom5020472 PMC4496681 25884116

